# A Comparison of the Socio-Behavioral-Metabolic Risk Profiles and Associated Factors for Chronic Diseases of Lifestyle in Urban and Rural Communities in Central South Africa

**DOI:** 10.3389/fpubh.2020.570676

**Published:** 2020-10-16

**Authors:** Sanet van Zyl, Francios C. van Rooyen, Gina Joubert, Willem H. Kruger, Corinna M. Walsh

**Affiliations:** ^1^Department of Basic Medical Sciences, Faculty of Health Sciences, University of the Free State, Bloemfontein, South Africa; ^2^Department of Biostatistics, Faculty of Health Sciences, University of the Free State, Bloemfontein, South Africa; ^3^Department of Community Health, Faculty of Health Sciences, University of the Free State, Bloemfontein, South Africa; ^4^Department of Nutrition and Dietetics, Faculty of Health Sciences, University of the Free State, Bloemfontein, South Africa

**Keywords:** non-communicable diseases (NCDs), chronic diseases of lifestyle (CDL), socio-behavioral-metabolic risk profiles, obesity, cardiovascular disease, hypertension, type 2 diabetes mellitus, community-based primary health care prevention

## Abstract

**Background:** The global escalating prevalence of lifestyle-related non-communicable diseases places a significant burden on health systems. Chronic diseases of lifestyle (CDL) are a group of diseases that share similar modifiable risk factors that can result in long-term disease processes. Considering the socio-behavioral-metabolic risk profiles of communities and risk factors predictive of the presence of CDL can assist in the development of focused and effective community-based prevention, intervention, and treatment programs for CDL.

**Aim:** To determine the socio-behavioral-metabolic risk profiles and identify associated factors for the following CDL: obesity, cardiovascular disease, hypertension, and type 2 diabetes mellitus in rural and urban communities in central South Africa.

**Methodology:** This cross-sectional study included adults aged 25–65 years in the rural Southern Free State and urban Mangaung. Social determinants, behavioral and metabolic risk factors, and inflammatory biomarkers for CDL were determined.

**Results:** In total, 575 rural (mean age: 42 years; 71% female) and 429 urban (mean age: 44 years; 76% female) participants were included in the study. More than 20% of participants in both communities reported being previously diagnosed with cardiovascular diseases; with reported hypertension and diabetes mellitus more prevalent among rural participants. Insufficient intake of fruit and vegetables, alcohol use, and high blood pressure were among the top five risk factors in both communities. Physical inactivity ranked among the top two risk factors in the urban community; while alcohol and tobacco use was significantly higher in the rural community. Fibrinogen was the most prevalent inflammatory marker in both communities (32.9 rural vs. 48.3% urban). High sensitivity C-reactive protein (Hs-CRP), only available for rural participants, was high with increased levels in more than 80% of participants. In both communities, being female, having high blood pressure and increased fibrinogen levels were associated with obesity.

**Conclusion:** This study illustrated the high prevalence of socio-behavioral-metabolic risk factors for CDL, and identified similarities and distinct differences in the risk profiles of rural and urban communities. Considering the CDL risk profiles of communities can assist in prioritizing health needs and contribute to the development of tailor-made community-based primary health care prevention, intervention, and health promotion programs.

## Introduction

Non-communicable diseases (NCDs), also called chronic diseases, are a group of conditions that account for millions of deaths globally each year (1). Current projections indicate a significant rise in NCDs among the ranks of the top 10 leading global causes of death by 2040, with NCDs mortality projected to be the leading cause of death in the African region by 2030 (2, 3). The World Health Organization's (WHO) country-specific profiles for NCDs (3) illustrate the escalating prevalence of modifiable lifestyle/behavioral risk factors in South Africa, that can lead to physiological and metabolic changes (obesity, high blood pressure, hyperglycemia, and hyperlipidemia) and result in chronic diseases of lifestyle (CDL), including hypertension, chronic lung diseases, cancer, and diabetes.

An increasing number of studies show strong evidence of the integral role that socio-demographic or background risk factors play in the multifactorial etiology of CDL, especially in developing countries. Moving from the conventional biomedical approach, to a holistic biopsychosocial approach that considers socio-demographic determinants, such as population aging and growth, low levels of education, poverty, inequitable access to health care, rapid urbanization, and adoption of a more westernized lifestyle, is required to address the escalating burden of CDL (1, 4–6).

In recent years, attention has also shifted from focusing only on adult lifestyle-related risk factors as a cause for maintaining the risk cycle for CDL to adopting a life-course approach that acknowledges the influence of early life experiences on adult health and mortality. It has become clear that chronic low-grade systemic inflammation is the root cause of many chronic diseases with evidence proposing links between early adversity (e.g., an unfavorable early environment), impairment of immune status, and susceptibility to CDL later in life (7, 8). The chronic low-grade inflammatory state is characterized by increased levels of inflammatory biomarkers that reflect inflammatory load, for example; high sensitivity C-reactive protein (Hs-CRP), fibrinogen, high density lipoprotein (HDL), white blood cells (WBC), and cortisol (9–12). Lifestyle interventions, including exercise, healthy diet, and sleep patterns, managing stress and a focus on overall well-being, may have multiple anti-inflammatory effects, including favorable long-term changes in biomarkers of inflammation that may lower the risk of CDL (4, 13). Health care professionals are encouraged to advocate for an anti-inflammatory lifestyle and purposefully address it at patient, community, and national levels to improve the health and well-being of populations (4).

The National Development Plan (NDP) 2015–2020 of the South African Department of Health, focuses on nine long-term health goals to improve the health and well-being of the South African population and to strengthen health systems (14). One of the envisioned goals is a significant reduction in the prevalence of chronic diseases in South Africa by 2030. Primary health care facilities are the first point of contact with health services. In South Africa, these facilities are dominated by lifestyle-related chronic diseases that create a significant burden on health systems, with hypertension and type 2 diabetes mellitus ranked first and fourth, respectively, on the list of the top 25 diagnoses made at primary health care facilities (15). Mayosi et al. (5) highlighted the escalating burden and emerging differences in CDL profiles in rural and urban communities in South Africa, and the need for improved community-based health promotion and care.

Assessing and understanding the health needs of different communities can contribute to focused, targeted primary health care prevention, intervention, and treatment programs for CDL in resource-constrained settings, and support the vision of the NDP to reduce the prevalence of chronic diseases in South Africa. Detailed information relating to the multifactorial risk profile and factors maintaining the risk cycle for CDL in rural and urban communities in the Free State, South Africa are still limited and therefore this study was envisioned. The study aimed to determine the socio-behavioral-metabolic risk profiles and inflammatory markers, and to identify clusters of associated risk factors for the following CDLs: obesity, cardiovascular disease (CVD), hypertension, and type 2 diabetes mellitus with CDL in rural and urban communities in the Free State.

## Methods

### Study Design, Population, and Sampling

This cross-sectional epidemiological study formed part of the “Assuring Health for All” in the Free State (AHA FS) research study that investigated how living in rural and urban areas affects a person's lifestyle, health, and well-being. The study was conducted in two community service delivery sites of the University of the Free State. The rural part of the study was conducted in the southern Free State district of Xhariep in the rural towns of Springfontein, Trompsburg, and Phillippolis that form part of the service area of the Free State Rural Development Partnership Programme (FSRDPP). Adult members, aged 25 to 64 years, from households (formal plots and squatter households) in surrounding township areas of the three rural towns were eligible to participate in the study. Trained field workers visited all eligible households in these areas to explain the purpose of the study and recruit participants for the study. The urban part of the study was conducted in the service area of the Mangaung University Community Partnership Programme (MUCPP) clinic and a stratified proportional cluster sample of the communities in this service area was selected for the study. After determining the number of plots in the MUCPP service area using a municipal map a stratified proportional cluster sample, stratified by area and formal plots and squatter households in open areas, were done. After selecting X and Y coordinates randomly, 100 starting points were selected and from each starting point five adjacent households were approached by field workers and informed about the study. In each household all adult household members (25–64 years) were eligible to participate in the study. Trained field workers visited all eligible households to explain the purpose of the study and recruit participants for the study.

### Data Collection Procedures and Laboratory Analysis

Data collection took place in community halls in the three rural towns of the Free State for the rural part of the study and in the MUCPP clinic for the urban part of the study. All participants were interviewed to complete questionnaires related to household socio-demographic, individual health, physical activity, and diet information. Language interpreters for Sesotho, Setswana, and isiXhosa-speaking participants assisted the researcher, where necessary. Senior dietetic students, under the supervision of lecturers from the Department of Nutrition and Dietetics at the University of the Free State (UFS), interviewed the participants and completed the questionnaires that focused on household socio-demographic information (e.g., age, gender, level of education, employment status, experienced stress) and health information that included lifestyle-related risk factors, family, and medication history.

Assessment of physical activity included a 24-h recall of all physical activities performed during the previous day, which included information on the time spent doing vigorous physical activity, moderate physical activity, walking activity, and sedentary periods. Frequency of activities that was not undertaken every day (e.g., gardening) was also determined. Using this information, the researchers calculated the physical activity level (PAL) for each participant, which were classified as: sedentary 1–1.39 PAL, low activity 1.4–1.59 PAL, active 1.6–1.89 PAL, and very active 1.9–2.5 PAL (16).

Anthropometric measurements were taken according to standard WHO procedures and included weight in kilograms (kg), height in centimeters (cm), and waist circumference (cm). Weight was determined using a Seca® (Germany) digital electronic foot scale, with participants in examination gowns, without shoes. Anthropometric indices included body mass index (BMI) (weight in [kg] divided by the square of the standing height [m^2^]), with overweight classified as a BMI ≥ 25 kg/m^2^ and obesity a BMI of ≥ 30 kg/m^2^ (17). The cut-off point for central obesity was a waist circumference of ≥88 cm in women and ≥102 cm in men (18).

In addition to anthropometric assessment, medical examinations were conducted by qualified medical professionals from the Department of Basic Medical Sciences of the Faculty of Health Sciences, UFS. Participants with urgent medical conditions were referred on the day of the medical examination, and participants could obtain results of biochemical tests and referral letters (where necessary) during follow-up visits. Blood pressure was measured during the clinical examination in the supine position with a DS-175, auto-inflate electronic blood pressure monitor (Nissei, Japan). Hypertension was defined as a systolic blood pressure of 140 mmHg or higher and/or a diastolic pressure of 90 mmHg or higher (19).

Fasting blood samples were obtained to determine biomedical risk factors as well as inflammatory markers. All analyses were performed by an accredited laboratory. Serum and plasma samples were prepared in the laboratory according to standard methods and stored at −80°C until analyses were performed. Complete full blood counts were performed using the Roche Sysmex XT 2000i analyzer® (Sysmex Sverige, Kungsbacka, Telefon). Blood specimens for the measurement of fasting venous plasma glucose were drawn into fluoride tubes. Samples were centrifuged within 4 h and fasting venous plasma glucose was measured using the glucose oxidase method on a Beckman LX20® auto-analyzer (Beckman Coulter, Fullerton, CA. Diabetes mellitus was defined as fasting plasma glucose value ≥ 7.0 mmol/L [126 mg/dl] and HbA1c levels were defined as prediabetes: 5.7–6.4% (39–47 mmol/mol) and diabetes: 6.5% or higher (48 mmol/mol or higher) (20, 21).

Fasting serum lipid levels were measured using enzymatic assay kits on a Beckman LX20® auto-analyzer (Beckman Coulter, Fullerton, CA). Raised blood lipids were defined as total cholesterol ≥ 200 mg/dl (5.2 mmol/l); low-density lipoprotein (LDL) cholesterol ≥ 100 mg/dl (2.59 mmol/L); triglycerides ≥ 150 mg/dl (1.7 mmol/L). Hs-CRP results were categorized as low risk: <1.0 mg/L, average risk (1.0–3.0 mg/L) or high risk (>3.0 mg/L) levels, as indicated by the American Heart Association and US Centers for Disease Control and Prevention recommendations (22). Raised total leucocytes was defined as ≥9.88 × 10^9^/L; fibrinogen ≥ 290 mg/dl. The ratio of NLR was determined with normal value ranging between 0.78 and 3.53 (23, 24).

### Statistical Analysis

Data were statistically analyzed by the Department of Biostatistics, UFS, using frequencies and percentages for categorical variables and means for numerical variables. Comparisons between rural and urban participants were done using contingency tables with chi-squared or Fisher's exact tests as appropriate. A level of significance was accepted as a two-tailed *P*-value < 0.05.

To identify factors for CDL, logistic regression models with each potential risk factor, age, and gender were fitted. Risk factors with *P*-value < 0.15 were included in the stepwise logistic regression models to identify significant associated risk factors. Stepwise logistic regression starting with all variables included in the model (i.e., backward elimination) was performed, using *P* = 0.05 as threshold for entry and exit. If a variable with multiple missings was not significant in the model, the model was run again excluding that variable. Odds ratios (OR) and 95% confidence intervals (95% CI) were used to present risk factors identified through this process for the following: CDL; obesity, hypertension, diabetes, and CVDs. Obesity was defined as a BMI of ≥30 kg/m^2^; hypertension as systolic blood pressure ≥140 mmHg and/or diastolic blood pressure ≥90 mmHg) or on medication to lower blood pressure; and diabetes mellitus as fasting plasma glucose value ≥7.0 mmol/L or with a history of diagnosis of diabetes. Reported history of CVDs included stroke, heart disease, angina, heart attack, and heart failure.

The socio-behavioral-metabolic risk factors and inflammatory biomarkers categorized and defined below were considered for inclusion in the univariate logistic regression models. Variables which were significant in the different models were selected and considered for the final multivariate logistic regression models.

Behavioral risk factors were defined as:

Physically inactivity (level 1: sedentary— <60 min of moderate to vigorous-intensity activity daily, and level 2: low activity— <150 min of moderate-intensity activity per week); unhealthy diet (insufficient intake of fruit and vegetables/low consumption of fruits and vegetables defined as a consumption of <5 total servings [400 g] of fruit and vegetables per day); tobacco use (currently smoke and formerly smoked); alcohol use (currently and formerly used).

Metabolic risk factors with categorical cut points were defined as follows:

Raised blood glucose (fasting plasma glucose value ≥7.0 mmol/L [126 mg/dl]); HbA1c (diabetic: 6.5% or higher [48 mmol/mol or higher]); high blood pressure (elevated systolic blood pressure ≥140 mmHg and/or diastolic blood pressure ≥90 mmHg); overweight (BMI ≥25 kg/m^2^) and obesity (≥30 kg/m^2^); increased waist circumference (men ≥102 cm [high risk]; women ≥ 88 cm [high risk]); raised blood lipids (total cholesterol ≥200 mg/dl [5.2 mmol/L]); LDL cholesterol ≥ 100 mg/dl (2.59 mmol/L); triglycerides ≥150 mg/dl (1.7 mmol/L).

Inflammatory biomarkers with categorical cut points were defined as follows:

Hs-CRP: average risk (1.0–3.0 mg/L) or high risk (>3.0 mg/L); total leucocytes (>9.88 × 10^9^/L); NLR ≥ 3.35; fibrinogen ≥ 290 mg/dl.

### Ethical Considerations

Ethics approval to conduct the AHA-FS study was obtained from the Ethics Committee of the Faculty of Health Sciences (UFS-ETOVS number 21/07), the Health Sciences Research Ethics Committee (HSREC) of UFS (UFS-HSD2017/1435), the Free State Department of Health and local municipalities before commencement of the study. In all communities, trained field workers recruited participants during home visits and the research team explained the project to participants before written informed consent was obtained from each participant.

## Results

### Study Population

A total number of 1,004 (575 rural and 429 urban) participants, between 25 and 65 years adhered to the study criteria and were included in the study. The mean age of the rural and urban participants was 47.2 years (71% females) and 44.4 years (76% females), respectively.

### Socio-Demographic Characteristics and Background Risk Factors of Study Participants

Results obtained through socio-demographic questionnaires, summarized in Table 1, revealed that a higher percentage of urban (19.9) vs. rural (15.4%) participants completed grade 11–12 at school level. Reported unemployment status was higher in the urban community (54.8%) than the rural community (24.2%). While 31.0% of urban participants reported that they experienced permanent stress, only 9.0% of rural participants reported similar levels of stress. Results obtained through individual health questionnaires are summarized in Table 1, indicated the high prevalence of hypertension, diabetes mellitus, and CDVs among rural and urban participants and their family members.

**Table 1:** Reported socio-demographic/background risk factors of rural and urban participants.

**Risk factor**	**Rural**			**Urban**			***P*-value for % difference**
	***N***	***n***	**%**	***N***	***n***	**%**	
**Age**	**575**			**429**			<0.01
25–29 years		34	5.9		51	11.9	
30–34 years		52	9.0		37	8.6	
35–39 years		67	11.7		64	14.9	
40–44 years		80	13.9		58	13.5	
45–54 years		158	27.5		125	29.2	
55–65 years		184	32.0		94	21.9	
**Gender**	**575**			**429**			0.08
Men		167	29.0		103	24.0	
Women		408	71.0		326	76.0	
**Marital status**	**546**			**403**			<0.01
Never married		110	20.1		137	34.0	
Married/Traditional marriage		182	33.3		120	29.8	
Living with a partner		73	13.4		40	9.9	
Widowed		104	19.1		58	14.4	
Separated		55	10.1		18	4.5	
Divorced		21	3.8		29	7.2	
Other		1	0.2		1	0.3	
**Medical history**							
Previously diagnosed with diabetes mellitus	**547**	60	11.0	**405**	30	7.4	0.06
Previously diagnosed with hypertension	**548**	346	63.1	**405**	195	48.2	<0.01
Previously diagnosed with the following CDVs:							
Stroke	**549**	36	6.6	**405**	21	5.2	0.33
Heart disease, angina, heart attack	**545**	88	16.2	**405**	68	16.8	0.79
Heart failure	**549**	6	1.1	**405**	19	4.7	<0.01
**Family history**							
Member previously diagnosed with diabetes mellites	**548**	145	26.6	**405**	134	33.1	0.03
Member previously diagnosed with hypertension	**544**	333	61.2	**405**	251	62.0	0.81
Member previously diagnosed with the following CDVs:							
Stroke	**543**	84	15.5	**405**	78	19.3	0.12
Heart disease, angina, heart attack	**542**	107	19.8	**405**	107	26.4	0.01
Heart failure	**546**	33	6.0	**405**	26	6.4	0.81
**Level of education**	**550**			**416**			<0.01
None		144	26.2		76	18.3	
Primary school education		173	31.5		153	36.8	
Secondary school education:							
Grade 8−10		145	26.4		100	24.0	
Grade 11–12		85	15.5		84	20.2	
Tertiary education		3	0.6		3	0.7	
**Employment status**	**575**			**429**			<0.01
Housewife by choice		14	2.4		3	0.7	
Unemployed		139	24.2		235	54.8	
Self-employed		9	1.6		4	0.9	
Full-time wage earner (receive a salary)		42	7.3		21	4.9	
Other (part-time job, piece job)		371	64.5		166	38.7	
**Experienced stress**	**539**			**405**			<0.01
Never		173	32.1		54	13.3	
Few periods of stress		173	32.1		129	31.9	
Several periods of stress		145	26.9		96	23.7	
Permanent stress		48	8.9		126	31.1	

*CDV, cardiovascular disease; N, number of participants*.

A large percentage of rural and urban participants (63.1 and 48.2%, respectively) reported having hypertension themselves, with just over 60% of participants in both study groups reporting a family history of hypertension. Self-reported diabetes mellitus was higher in the rural than urban (11.0 vs. 7.4%) participants, with more than a quarter of participants (26.0 rural and 33.1% urban) reporting a family history of diabetes mellitus. Twenty-four percent and 26.7% of rural and urban participants, respectively, reported being previously diagnosed with CVDs (stroke, heart disease, angina, heart attack and heart failure) and a high reported family history was also observed among both study populations (rural 41.3 and urban 52.1%).

The high prevalence of behavioral and metabolic risk factors among the rural and urban study populations are illustrated in Table 2. Significant differences between rural and urban participants were observed in tobacco use (*P* < 0.01), alcohol use (*P* < 0.01), PAL (*P* < 0.01), total cholesterol levels (*P* < 0.01), and high blood pressure (*P* < 0.01). The different behavioral and metabolic risk factors were ranked, and Table 3 illustrates distinct differences between the risk profiles of rural and urban communities. Insufficient intake of fruit and vegetables was the leading risk factor in both communities. Tobacco and alcohol use ranked higher in the rural community. Physical inactivity ranked among the top two risk factors in the urban community followed by high blood pressure. More than half of participants in both communities were either overweight or obese.

**Table 2:** Summary of behavioral, metabolic and inflammatory risk factors of rural and urban participants.

**Risk factor**	**Parameter**	**Rural**			**Urban**			***P*-value for % difference**
		***N***	***n***	**%**	***N***	***n***	**%**	
		**575**			**429**			
**BEHAVIORAL RISK FACTORS**								
Tobacco use	Currently smoke & formerly smoked	548	322	58.8	405	130	32.1	<0.01
Insufficient intake of fruit and vegetables	Consumption <5 servings (400 g) of fruit and vegetables per day	550	530	96.4	418	410	98.1	0.11
Alcohol use	Currently intake and formerly used	546	437	80.0	404	219	54.2	<0.01
Physical inactivity	Level 1 (i.e., sedentary: <60 min of moderate to vigorous-intensity activity daily) and Level 2 (i.e., low activity: <150 min of moderate-intensity activity per week)	550	150	27.3	415	276	66.5	<0.01
**METABOLIC RISK FACTORS**								
Elevated blood lipids (total cholesterol)	High risk: ≥240 mg/dl (≥6.22 mmol/L)	552	75	14.2	415	17	4.1	<0.01
High blood glucose	≥7.0 mmol/L (126 mg/dl)	544	43	7.9	411	18	4.4	0.03
HbA1c	Diabetes: 6.5% or higher (48 mmol/mol or higher)	548	53	9.7	415	25	6.0	0.04
High blood pressure	Systolic blood pressure of 140 mmHg or higher and/or a diastolic pressure of 90 mmHg or higher	563	382	67.9	413	235	56.9	<0.01
Body mass index	Overweight and obese BMI ≥25.00	555	295	53.2	419	227	54.2	0.75
Waist circumference	Men ≥102 cm (high risk)/Women ≥88 cm (high risk)	547	323	59.1	418	223	53.4	0.08
**INFLAMMATORY RISK FACTORS**								
Total leucocytes	(>9.88 × 10^9^/L)	543	71	13.1	417	18	4.3	<0.01
Neutrophils	(>7.5 × 10^9^/L)	544	25	4.6	417	6	1.4	0.01
Lymphocytes	(>4.0 × 10^9^/L)	544	4	7.7	417	7	1.7	<0.01
Monocytes	(>1.8 × 10^9^/L)	543	35	6.5	417	25	6.0	0.78
Neutrophil:lymphocyte ratio	≥3.53	543	29	5.3	417	17	4.1	0.36
Fibrinogen	≥290 mg/dl	520	171	32.9	389	188	48.3	<0.01

**Table 3:** Ranked behavioral and metabolic risk factors of rural and urban participants.

**Rural**	**Percentage (%)**	**Ranking**	**Percentage (%)**	**Urban**
Insufficient intake of fruit and vegetables	96.4	**1**	98.1	Insufficient intake of fruit and vegetables
Alcohol use	80.0	**2**	66.5	Physical inactivity
High blood pressure	67.9	**3**	56.9	High blood pressure
Increased waist circumference	59.1	**4**	54.2	Alcohol use
Tobacco use	58.8	**5**	54.2	Body mass index
Body mass index	53.2	**6**	53.4	Increased waist circumference
Physical inactivity	27.3	**7**	32.1	Tobacco use
Elevated blood lipid levels	14.2	**8**	6.0	Elevated HbA1c levels
Elevated HbA1c levels	9.7	**9**	4.4	Elevated blood glucose levels
Elevated blood glucose levels	7.9	**10**	4.1	Elevated blood lipid levels

**Figure 1:**
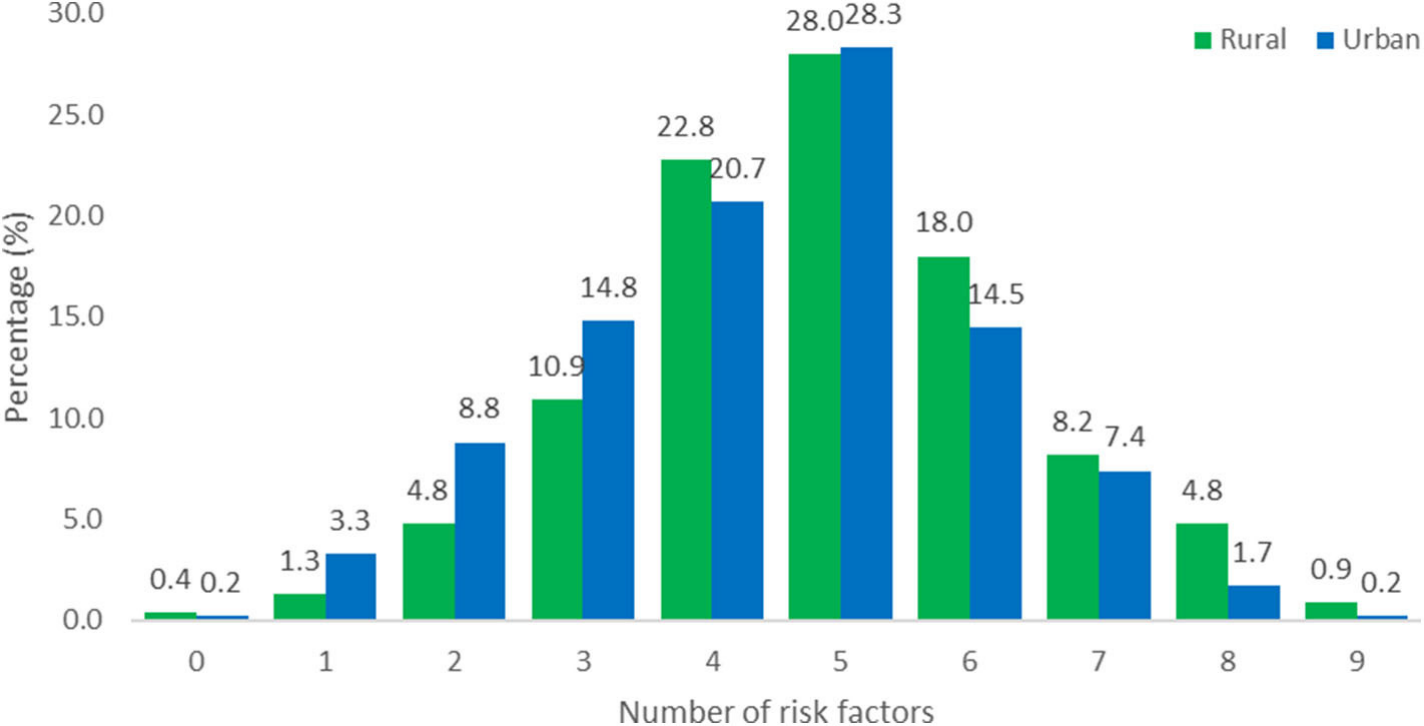
Total number of behavioral and metabolic risk factors for rural (*N* = 561) and urban (*N* = 420) participants. The data of 14 rural participants and 9 urban participants were incomplete.

The number of risk factors in the two populations were calculated and illustrated in Figure 1. Due to the presence of missing information it was decided to include all participants with information for seven or more of the 10 behavioral and metabolic risk factors in the calculations. Of the 575 rural participants, the data of 561 were included and of the 429 urban participants, the data of 420 were included (Table 3). For participants with information for 7, 8 or 9 of the risk factors, the number of risk factors present were calculated proportional to the number of risk factors with known information and rounded down to a value between 0 and 10. For example 1 risk factor present out of 7 with known information was calculated as 1/7^*^10 = 1.4 and reported as 1.

Markers of inflammation are summarized in Table 2. Hs-CRP results (average risk: 1.0–3.0 mg/L and high risk: >3.0 mg/L) were only available for rural participants, with average and high levels observed in 88.5% of rural participants. Fibrinogen was the most prevalent inflammatory marker in both communities with 32.9% of rural participants and 48.3% of urban participants having elevated levels. Significant differences for total leucocytes (*P* < 0.01), lymphocytes (*P* < 0.01), and fibrinogen (*P* < 0.01) were observed between rural and urban participants, with more rural participants presenting with elevated leucocytes (13.1 rural vs. 4.3% urban) and lymphocytes (7.7 rural vs. 1.7% urban) and more urban participants with elevated fibrinogen. The least prevalent inflammatory marker in both communities was elevated neutrophils (4.6 rural vs. 1.4% urban).

In addition to the comparison of the socio-behavioral-metabolic risk factors and inflammatory biomarkers for CDL in rural and urban participants, univariate analysis was performed to identify risk factors associated with obesity, hypertension, diabetes, and CVDs, results are illustrated in Table 4. The number of participants excluded due to missing values include 20 rural and 17 urban participants for obesity, 9 rural and 7 urban participants for hypertension, 29 rural and 17 urban for diabetes, 26 rural and 24 urban participants for cardiovascular disease.

**Table 4:** Significance of individual risk factors for chronic diseases of lifestyle (CDL).

	**Obesity**		**Hypertension**		**Diabetes**		**Cardiovascular diseases**	
	**Rural**	**Urban**	**Rural**	**Urban**	**Rural**	**Urban**	**Rural**	**Urban**
**SOCIO-DEMOGRAPHIC/BACKGROUND RISK FACTORS**								
Age	0.56	**0.04**	**<0.01**	**<0.01**	**0.03**	**0.01**	**0.06**	**0.03**
Gender	**0.01**	**<0.01**	0.15	**0.02**	0.86	0.64	0.40	0.64
Level of education	**<0.01**	**0.06**	0.38	0.99	0.55	0.32	**0.09**	0.76
Experienced stress	0.23	0.26	**0.02**	0.42	0.53	0.32	**<0.01**	**<0.01**
**BEHAVIORAL RISK FACTORS**								
Physically inactivity	0.66	0.80	0.54	**0.07**	**0.03**	0.95	0.19	0.43
Unhealthy diet	0.56	0.86	0.30	0.92	**0.01**	**0.06**	0.24	0.55
Tobacco use	**<0.01**	**<0.01**	0.97	0.84	**0.01**	**<0.01**	**0.07**	0.62
Alcohol use	**0.01**	0.25	0.90	0.66	**0.06**	**0.08**	0.58	**0.12**
**METABOLIC RISK FACTORS**								
Raised fasting blood glucose	**<0.01**	**<0.01**	**0.13**	**0.03**	-	-	0.78	**0.04**
HbA1c	**<0.01**	0.98	**0.03**	0.17	**<0.01**	**<0.01**	**0.14**	**0.27**
High blood pressure	**0.01**	**<0.01**	-	-	0.15	**0.03**	0.26	**<0.01**
Overweight and obesity	-	-	**0.01**	**<0.01**	**<0.01**	**<0.01**	0.41	0.34
Increased waist circumference	**<0.01**	**<0.01**	**<0.01**	**<0.01**	**<0.01**	**0.01**	0.26	0.21
Raised total cholesterol	0.92	0.45	**0.04**	0.16	0.23	0.97	0.19	0.64
LDL cholesterol	**0.09**	**0.07**	0.29	0.25	0.48	0.73	0.21	0.61
Triglycerides	**<0.01**	0.57	**0.14**	**0.04**	**0.01**	0.62	0.91	0.17
**INFLAMMATORY BIOMARKERS**								
Hs-CRP	**<0.01**	ND	0.47	ND	0.23	ND	0.65	ND
Leucocytes	0.52	0.17	0.45	0.61	**0.06**	0.77	0.41	**0.04**
Neutrophil	0.58	0.19	0.83	0.39	0.42	0.48	0.25	**0.09**
Neutrophil:lymphocyte ratio	**<0.01**	0.71	0.89	0.57	0.17	0.80	0.43	0.97
Fibrinogen	**<0.01**	**<0.01**	0.94	0.78	0.26	0.16	0.21	0.27

*Risk factors with p-value <0.15 were included in the stepwise logistic regression models to identify significant (p-value <0.05) associated risk factors as displayed in Table 5*.

*Note missing values: Obesity (rural 20, urban 17); Hypertension (rural 9, urban 7), Diabetes (rural 29, urban 17), Cardiovascular disease (rural 26, urban 24)*.

*ND, not done*.

Results of the multivariate logistic regression models are indicated in Table 5.

**Table 5:** Significant socio-behavioral-metabolic risk factors, inflammatory biomarkers associated with the presence of obesity, cardiovascular disease, hypertension, and diabetes mellitus in a rural and urban sample.

**Variables**		**Rural**		**Urban**	
		**Odds ratio (95% confidence interval)**	^*^P-value	**Odds ratio (95% confidence interval)**	^*^P-value
**OBESITY**					
Sex	Female vs. male	3.90 [2.35;6.48]	<0.01	8.64 [4.14;18.04]	<0.01
Smoke	Yes vs. No	0.25 [0.16;0.40]	<0.01	0.41 [0.23;0.72]	<0.01
High blood pressure	Yes vs. No	1.72 (1.01;2.94]	0.05	4.54 [2.60;7.93]	<0.01
High blood glucose levels	Yes vs. No			3.41 [1.27;9.13]	0.02
Elevated triglycerides	Yes vs. No	2.95 [1.86;4.66]	<0.01		
Average/high-risk Hs-CRP	Yes vs. No	2.59 [1.20;5.60]	0.02		
Elevated fibrinogen	Yes vs. No	2.04 [1.28;3.24]	<0.01	2.11[1.27;3.49]	<0.01
**HYPERTENSION**					
Age(years)	55–65 vs. 25–34	11.82 [5.40;25.84]	<0.01	19.16 [7.86;46.74]	<0.01
Age (years)	45–54 vs. 25–34	6.20 [3.05;12.60]	<0.01	7.74 [3.96;15.14]	0.04
Age (years)	35–44 vs. 25–34	1.93 [1.04;3.56]	0.01	3.03 [1.61;5.68]	0.02
Stress	Never vs. permanent stress	0.27 [0.08;0.95]	<0.01		
Stress	Few periods of stress vs. permanent stress	0.40 [0.11;1.44]	0.21		
Stress	Several periods of stress vs. permanent stress	0.77 [0.21;2.88]	0.18		
BMI				2.19 [1.08;4.41]	0.03
Waist circumference				2.44 [1.22;4.90]	0.01
HbA1c	Diabetes (Yes vs. No)	10.40 [1.37;78.98]	0.02		
**DIABETES**					
Age(years)	55–65 vs. 25–34			8.84 [1.92;40.66]	<0.01
Age (years)	45–54 vs. 25–34			7.32 [1.61;33.30]	0.02
Age (years)	35–44 vs. 25–34			2.23 [0.44;11.41]	0.25
Smoke	Yes vs. No			0.23 [0.08;0.60]	<0.01
Physical inactivity	1 vs. 2	1.84 [1.01;3.38]	0.05		
Increased waist circumference	Yes vs. No	4.10 [1.91;8.77]	<0.01		
Elevated triglycerides	Yes vs. No	2.14 [1.19;3.82]	0.01		
Elevated leucocytes	Yes vs. No	2.33 [1.15;4.67]	0.02		
**CARDIOVASCULAR DISEASES**					
Stress	Never vs. permanent stress	1.90 [0.88;4.11]	0.05	0.75 [0.31;1.82]	0.86
Stress	Few periods of stress vs. permanent stress	2.14 [0.98;4.67]	0.01	0.30 [0.13;0.66]	<0.01
Stress	Several periods of stress vs. permanent stress	0.74 [0.35;1.56]	<0.01	1.75 [0.93;3.30]	<0.01
Education	None vs. tertiary education	2.91 [1.34;6.30]	0.04		
Education	Primary school vs. tertiary education	2.03 [1.12;3.67]	0.31		
Education	Grade 8–12 vs. tertiary education	1.24 [0.73;2.11]	0.12		
High blood pressure				2.84 [1.39;5.78]	<0.01
Glucose levels	Diabetes (Yes vs. No)			2.24 [1.03;4.88]	0.04
Elevated leucocytes	Yes vs. No			3.17 [1.09;9.21]	0.03

* *P <0.05*.

*Note missing values*:

*Obesity: 85 rural and 57 urban observations were excluded due to missing values for the response or explanatory variables Hypertension: 50 rural and 8 urban observations were excluded due to missing values for the response or explanatory variables*.

*Diabetes: 66 rural and 19 urban observations were excluded due to missing values for the response or explanatory variables*.

*Cardiovascular diseases: 31 rural and 17 urban observations were excluded due to missing values for the response or explanatory variables*.

Multivariate logistic regression analysis showed that in the rural sample, being female, having high blood pressure, increased triglycerides, and increased levels of the inflammatory biomarkers Hs-CRP and fibrinogen were positively associated with **obesity**. In the urban sample, being female, having high blood pressure, high blood glucose, and fibrinogen levels were positively associated with being obese. As expected, smoking was negatively associated with obesity in rural (OR 0.25; *P* < 0.01) and urban (OR 0.42; *P* < 0.01). Age advancement was associated with the presence of **hypertension** in both rural and urban areas. The probability of having hypertension in the rural area was higher with increased reported stress levels, while high HbA1c levels increased the odds of having hypertension 10.40 (95% CI 1.37;78.98) times. In this sample, residing in an urban area, being overweight and obese were positively associated with the presence of hypertension.

In this study, adults living in an urban area had increased odds of **diabetes mellitus** with increased age; while increased waist circumference was positively associated with diabetes mellitus in rural (OR 4.10; *P* < 0.01) areas. Based on the OR, adults living in a rural area, with a sedentary/low activity lifestyle, increased triglycerides, and leucocytes had increased odds of having diabetes. Adults residing in a rural area, with no education were almost three times (OR 2.9, 95% CI 1.34; 6.30) more likely to have **CVD**s; in terms of the association between CVD and levels of stress, results were unexpected. Possible reasons for this will be included in the discussion. Urban participants with hypertension (OR 2.84, 95% CI 1.39; 5.78), elevated glucose (OR 2.24, 95% CI 1.03; 4.88), and leucocytes (OR 3.17, 95% CI 1.09; 9.21) were more likely to have CVDs.

## Discussion

Statistics South Africa (Stats SA) has reported an increase in deaths due to cardiovascular diseases since 2009 in South Africa, with the highest percentage of these deaths (57.4%) observed in 2016 (25). At that time, hypertension (ranked third), cerebrovascular disease (fourth), and diabetes mellitus (fifth) were among the 10 leading underlying natural causes of death in the Free State province. The social determinants of health and diseases cannot be ignored when looking at the mentioned deaths as studies concluded that the risk factors included several societal factors (26, 27). Identifying and addressing the major social determinants of health, forms an integral part of the South African National Department of Health's Primary Health Care Re-engineering Strategy (28). This study observed distinct differences between social determinants of CDL in rural and urban communities in the Free State. Although a higher percentage of urban participants had completed secondary school education, reported unemployment status was higher in the urban study population (54.8%) than in the rural group (24.2%). Almost a third of urban participants vs. 9% of rural participants reported that they experienced permanent stress which may be related to unemployment status.

The high prevalence of reported cardio-metabolic diseases among rural and urban participants and their family members in this study illustrated the intra- and intergenerational burden of CDL in these communities. Although prevalence of chronic diseases was high in both groups, a higher percentage of rural participants reported being diagnosed with hypertension and diabetes. The findings of the present study are supported by those reported in the South African National Health and Nutrition Examination Survey (SANHANES) by Shisana et al. (29), who showed that at that time the Free State province had the highest prevalence of self-reported high blood pressure (45.8%), high blood sugar (26.7%), heart disease (28.7%), and stroke (14.5%), with an increase in self-reported high blood pressure and stroke observed between 2003 and 2012. The findings of the current study further highlight important urban and rural differences.

A study undertaken among older adults from 2007 to 2010 in six low- and middle-income countries (China, Ghana, India, Mexico, Russian Federation and South Africa) by Wu et al. (30) ranked seven risk factors for CDL (inadequate vegetable and fruit intake, low PALs, smoking, frequent alcohol use, high blood pressure, obesity, central obesity) and revealed that South Africa had the highest prevalence for low PALs (59.7%) and obesity (45.2%) across the six countries. Similarly, the present study revealed that insufficient intake of fruit and vegetables was the leading risk factor for CDL in both the urban and rural study populations. The high prevalence of food insecurity in the study population may have been responsible for this, with 73.2 and 87.4% of the current rural and urban families, respectively, classified as having a high risk of food insecurity (31). Low intake of fruits and vegetables has been reported in other South African studies, such as in a rural Limpopo community, where Maimela et al. (32) reported that 88.6% of participants had a low daily intake of fruit and vegetables. A contributing factor for this observation in our rural study population could be the distances that these participants need to travel on foot to purchase fruit and vegetables (83 rural vs. 54% urban) (31). In addition, the transition to an unhealthier “Westernized” diet in South Africa is often characterized by low intake of fresh fruit and vegetables (33, 34).

As confirmed in the current study, urban dwellers are more likely to experience a sedentary lifestyle with decreased PALs (35). Physical inactivity was ranked among the top two risk factors for CDL in our urban community (66.5%) and ranked seventh (27.3%) in the rural community. Several studies have illustrated an inverse relationship between physical activity and the prevalence of CDL such as hypertension and diabetes mellitus (29), while regular physical activity has been shown to strengthen resilience to stress.

The SANHANES (29) found that 50.4% of Free State participants smoked tobacco in 2012; this is much higher than the national average of 32.8%. In the current study, significant differences were observed between tobacco use of urban and rural participants (58.8 vs. 32.1%; *P* < 0.01). Tobacco use ranked among the top five risk factors in the rural community but ranked much lower (seventh) in the urban community. In addition to the high prevalence of smoking reported in the rural study population, reported alcohol use ranked second in the rural study population (80.0 rural vs. 54.2% urban, *P* < 0.01). The recent South African Demographic and Health Survey (36) revealed a slight increase in the national prevalence of men consuming alcohol from 58 (1998) to 61% (2016), while the prevalence among women remained unchanged (26%).

In the present study, high blood pressure ranked among the top three risk factors in both communities, with prevalence levels of 67.9 and 56.9% among rural and urban participants, respectively. High prevalence of hypertension was also observed in the South African leg of the Prospective Urban Rural Epidemiological (PURE) Study (37), where a slightly higher prevalence of hypertension was seen in their urban (74.0) vs. rural (71.8%) study population. Concerning results of the South Africa Demographic and Health Survey (SADHS) (36, 38) indicated that of the 46% South African women and 44% men who had hypertension, more than 80% of women and 87% of men presented with uncontrolled hypertension. Although 11.0 and 7.4% (rural and urban participants, respectively) in the current study reported being diagnosed with diabetes, elevated HbA1c, levels that provide a longer-term view of glycemic status, was observed in 9.7 rural vs. 6.0% urban participants. These results reflect poorly controlled or undiagnosed diabetes.

A high prevalence of overweight/obesity and waist circumference is linked to the high prevalence of metabolic disorders such as hypertension and type 2 diabetes mellitus (29). In both the study populations of the current study, overweight/obesity ranked fourth and sixth among the 10 behavioral and biological/metabolic risk factors observed in this study. More than half of rural and urban participants (53.2 and 54.2%) were either overweight or obese and 59.1% of rural participants (20.3 men vs. 75.5% women) and 53.4% of urban participants (6.0 men vs. 68.0% women) had waist circumference levels above the cut-off values. The prevalence of overweight or obesity among females living in urban and rural settings (66.1 and 65.6%, respectively) in this study corresponds with findings of the SADHS (36) that reported that 68.4 and 66.1% females, living in urban and rural settings respectively, were either overweight or obese. In the current study, as in other South African surveys (29, 36), the prevalence of overweight and obesity was much lower among men than women.

Obesity is associated with chronic low-grade inflammation, and markers of inflammation such as CRP, TNF-α, and IL-6 have been reported to correlate positively with adipocyte size (39). Lifestyle-related risk factors such as physical inactivity, poor diet and emotional stress are considered to be triggers that can cause activation of the immune system and consequently the hypothalamic pituitary adrenal stress response system, increasing the risk for obesity and chronic diseases such as CVD and diabetes mellitus (8, 11, 40, 41). This study confirmed a high prevalence of inflammation in both urban and rural participants that could be attributed to the higher cumulative risk effects of multiple risk factors. More than half of both urban and rural participants had five or more risk factors present for CDL. Almost 90% of rural participants had high Hs-CRP levels that have been associated with lifestyle risk factors such as smoking and physical inactivity (42) and correlate positively with BMI (43). Significant differences (*P* < 0.01) were observed between the study populations for the following inflammatory markers; total leucocytes levels (13.1% rural vs. 4.3% urban), lymphocytes (7.7% rural vs. 1.7% urban) and fibrinogen (32.9% rural vs. 48.3% urban). An elevated leucocyte count is a strong independent risk factor for coronary heart disease morbidity and mortality, while NLR can also be used as a predicative tool for adverse cardiac events in diabetic patients with CVDs (44). Elevated fibrinogen levels were the highest-ranked inflammatory marker in both communities. The Emerging Risk Factors Collaboration group (45), investigated the value of CRP or fibrinogen in CVD prediction and found that one additional CDV event over a period of 10 years could be prevented with additional assessment of CRP or fibrinogen in patients at intermediate risk for a CDV event after initial screening of conventional risk factors. Chen and Lacey (7) used CRP and fibrinogen to indicate adult inflammation in a 1958 British birth cohort and illustrated that adverse childhood experiences, related to socio-economic and health behavioral factors, were associated with inflammation in mid-life.

In a community-based study in Shanghai, Zhang et al. (46) reported that hypertension, diabetes, and dyslipidemia were positively associated with obesity. In our study, multivariate logistic regression analysis revealed that in both the rural and urban sample, obesity was also associated with having hypertension. In addition, obesity was positively associated with being female and elevated fibrinogen levels. In the rural sample, obesity also increased the odds of having increased triglyceride and Hs-CRP levels, while in the urban sample, high blood glucose was positively associated with obesity. A community-based cross-sectional Ethiopian study (47), reported that obesity was positively associated with daily intake of alcohol; while our study found that former and current smoking was negatively associated with obesity in both the urban (OR 0.42; *P* < 0.01) and rural (OR 0.25; *P* < 0.01) study populations.

In terms of hypertension, multivariate logistic regression analysis showed that age advancement was more likely to be associated with the presence of hypertension in both rural and urban areas; a finding also observed in the earlier South African Adult Demographic and Health Survey (48) and a recent African based study (49). In addition, we found that the probability of having hypertension in the rural area was higher with increased reported stress levels, while high HbA1c levels increased the odds of having hypertension 10.40 (95% CI 1.37; 78.98) times. The current study also found that increased waist circumference and BMI ≥ 25.0 kg/m^2^ were positively associated with the presence of hypertension. Similar observations were made by Zekewos et al. (49) who reported that age advancement, BMI (≥25.0 kg/m^2^), and central obesity (waist-to-height ratio ≥0.50) were positively associated with hypertension. Mbouemboue and Ngoufack (50) also reported that in a low-resource African setting, age, overweight and high serum triglyceride level were identified as independent factors predicting hypertension. The observation of Mahmood et al. (51) that in addition to age, education was an independent risk factor of hypertension for urban participants living in India, was not confirmed in the current study; however, adults residing in a rural area, with higher education levels were three times more likely to have CVDs; this can possibly be attributed to higher employment and income levels that can lead to unhealthy eating habits due to a higher intake of fast foods (6). Urban participants with hypertension (OR 2.84, 95% CI 1.39; 5.78), high glucose levels (OR 2.24, 95% CI 1.03; 4.88), and elevated leucocytes (OR 3.17, 95% CI 1.09; 9.21) were more likely to have CVDs. The logistic regression related to CVD and stress delivered unexpected results. The authors acknowledge that categorizing stress as “never,” “few periods,” and “several periods” may have been confusing to participants. Answers to questions related to concrete variables such as age and level of education are more easily answered by participants than variables that are more fluid, such as one's experience of stress.

In terms of diabetes, for participants living in a rural area, being physically inactive, having increased waist circumference, elevated triglycerides and leucocytes levels emerged as important associated risk factors of diabetes, while in an urban area advanced age was associated with diabetes. Vijayakumar et al. (52) reported that advanced age and the presence of central obesity were important risk factors for type 2 diabetes mellitus in an Indian study. As part of lifestyle management, the American Diabetes Association (ADA) (53) recommends focused goal-based behavioral lifestyle intervention programs for prediabetes patients, that includes 7% weight loss and physical activity (150 min of physical activity, similar in intensity to brisk walking, per week).

Many governments are not keeping pace with the growing burden of lifestyle-related chronic diseases and associated demands on health services. Baird et al. (54) and Mikkelsen et al. (55) have emphasized the importance of adopting a life course approach that recognizes the opportunity to promote health, prevent and control NCDs at key stages throughout the life course, from preconception through to adulthood. Focusing on key life stages throughout the life course of the individual and implementing targeted lifestyle modifications and awareness programs can reduce the prevalence of prioritized risk factors for communities identified in this study, and break the risk cycle and the current intergenerational transmission of CDL in communities.

The authors acknowledge the following limitations of the study. Firstly, more females than males participated in the study, mostly because more males are employed laborers and therefore not available to participate on weekdays that the study was conducted and therefore, we acknowledge that the study population was not completely representative of the target population. We further acknowledge that not all relevant psychosocial factors (e.g., household composition, social support, depressive symptoms) were assessed or reported on in this publication. The exclusion of participants due to missing values occurred and is acknowledge by the authors as a limitation of the study, furthermore, we acknowledge that multiple testing took place due to the numerous variables involved and spurious results may occur.

## Conclusion and Recommendations

This study aimed to provide a better understanding of the health needs of urban and rural communities in the Free State with the aim of contributing to focused, targeted primary health care prevention and intervention programs for CDL, supporting the vision of the NDP to reduce the prevalence of chronic diseases in South African communities. The WHO has emphasized the importance of developing a holistic, integrated approach that targets the major common risk factors for CDL in the most cost-effective way to prevent and control CDL in communities.

Various studies have confirmed that a healthy lifestyle is associated with lower concentrations of inflammatory markers. Gaesser et al. (13) indicated that exercise and dietary factors, including dietary fiber, fruits (especially berries), omega-3 unsaturated fatty acids, antioxidant vitamins E and C and zinc could reduce inflammatory markers. Johannsen et al. (39) found that aerobic exercise not only reduced total WBC and neutrophil count in a group of overweight/obese women, but was particularly beneficial for those with low-grade systemic inflammation.

Based on the findings of this study, the researchers recommend:

- Focused and tailor-made community-based primary health care prevention [that include early screening toolkits and important point of care devices that can contribute to immediate identification of critical biochemical markers (HbA1c, Hs-CRP, fibrinogen)] and intervention programs that prioritize the identified multi-factorial etiology of CDL and related health needs in the different communities.
- Using the proposed approach to strengthen health and wellness educational programs in the different communities, with emphasis on the benefits of a healthy early environment and anti-inflammatory lifestyle throughout the individual's life course.
- Incorporating the following essential components in community-based chronic disease learning modules for undergraduate Health Professions programs:
  - °Contextual understanding of the multi-factorial etiology of CDL in different communities with emphasis on the different determinants (social, cultural, behavioral, biological, psychological, economic, and environmental) that drive CDL disease processes, at individual and collective levels in the communities.
  - °Promote a holistic integrated primary health care approach that focuses on the complementary roles of different health professionals involved in the care of patients with CDL in order to reduce the prevalence and associated prioritized risk factors of these diseases in the communities.

## Data Availability Statement

The raw data supporting the conclusions of this article will be made available by the authors, without undue reservation.

## Ethics Statement

The studies involving human participants were reviewed and approved by Ethics Committee of the Faculty of Health Sciences (UFS-ETOVS number 21/07) Health Sciences Research Ethics Committee (HSREC) of UFS (UFS-HSD2017/1435). The patients/participants provided their written informed consent to participate in this study.

## Author Contributions

SZ were also responsible for the protocol and manuscript preparation, data collection, and data interpretation. GJ and FR were responsible for the data analysis. CW was the principle investigator. All authors were involved in the protocol and publication planning, writing, editing, and finalization.

## Conflict of Interest

The authors declare that the research was conducted in the absence of any commercial or financial relationships that could be construed as a potential conflict of interest.

## Abbreviations

CDL: chronic diseases of lifestyle
CVD: cardiovascular disease
Hs-CRP: high sensitivity C-reactive protein
NCD: non-communicable disease
NDP: national development plan
PAL: physical activity level.

## References

[1] World Health Organization (WHO) Non-communicable diseases [Internet]. Geneva (2018). Available online at: https://www.who.int/en/news-room/fact-sheets/detail/noncommunicable-diseases (accessed May 27, 2020).

[2] Foreman KJ Marquez N Dolgert A Fukutaki K Fullman N McGaughey M. Forecasting life expectancy, years of life lost, and all-cause and cause-specific mortality for 250 causes of death: reference and alternative scenarios for 2016–40 for 195 countries and territories. Lancet. (2018) 392:2052–90. 10.1016/S0140-6736(18)31694-5 30340847 PMC6227505

[3] World Health Organization (WHO) WHO global health estimates 2016: deaths by cause, Age, Sex, by country and by region, 2000–2016 [Internet]. Geneva (2018). Available online at: https://www.who.int/healthinfo/global_burden_disease/estimates/en/index1.html (accessed May 27, 2020).

[4] Bennett JM Reeves G Billman GE Sturmberg JP. Inflammation–nature's way to efficiently respond to all types of challenges: implications for understanding and managing “the epidemic” of chronic diseases. Front Med. (2018) 5:316. 10.3389/fmed.2018.00316 30538987 PMC6277637

[5] Mayosi BM Flisher AJ Lalloo UG Sitas F Tollman SM Bradshaw D. The burden of non-communicable diseases in South Africa. Lancet. (2009) 374:934–47. 10.1016/S0140-6736(09)61087-4 19709736

[6] Steyn NP Labadarios D Nel JH. Factors which influence the consumption of street foods and fast foods in South Africa—a national survey. Nutr J. (2011) 10:104. 10.1186/1475-2891-10-104 21967754 PMC3222608

[7] Chen M Lacey RE. Adverse childhood experiences and adult inflammation: findings from the 1958 birth cohort. Brain Behav Immun. (2018) 69:582–90. 10.1016/j.bbi.2018.02.007 29458198

[8] Barbaresko J Koch M Schulze MB Nöthlings U. Dietary pattern analysis and biomarkers of low-grade inflammation: a systematic literature review. Nutr Rev. (2013) 71:511–27. 10.1111/nure.12035 23865797

[9] Calder PC Ahluwalia N Albers R Bosco N Bourdet-Sicard R Haller D. A consideration of biomarkers to be used for evaluation of inflammation in human nutritional studies. Br J Nutr. (2013) 109 (Suppl 1):S1–34. 10.1017/S0007114512005119 23343744

[10] Estampador AC Franks PW. Genetic and epigenetic catalysts in early-life programming of adult cardiometabolic disorders. Diabetes Metab Syndr Obes. (2014) 7:575–86. 10.2147/DMSO.S51433 25489250 PMC4257022

[11] Bosma-den Boer MM Van Wetten M-L Pruimboom L Chronic inflammatory diseases are stimulated by current lifestyle: how diet, stress levels and medication prevent our body from recovering. Nutr Metab (Lond). (2012) 9:32 10.1186/1743-7075-9-32 22510431 PMC3372428

[12] Rhen T Cidlowski JA. Anti-inflammatory action of glucocorticoids-new mechanisms for old drugs. N Engl J Med. (2005) 353:1711–23. 10.1056/NEJMra050541 16236742

[13] Gaesser GA Angadi SS Ryan DM Johnston CS Lifestyle measures to reduce inflammation. Am J Lifestyle Med. (2012) 6:4–13. 10.1177/1559827611411646

[14] Department of Health Strategic Plan 2015/16–2019/20 A long and healthy life for all South Africans. Available online at: http://www.health.gov.za/index.php/2014-03-17-09-09-38/strategic-documents/category/229-2015str (accessed May 08, 2019).

[15] Mash B Fairall L Adejayan O Ikpefan O Kumari J Mathee S. A morbidity survey of south african primary care. PLoS ONE. (2012) 7:e32358. 10.1371/journal.pone.0032358 22442666 PMC3306367

[16] Frary CD Johnson RK Energy. In: Mahan LK Escott-Stump S editors. Krause's Food, Nutrition and Diet Therapy. 11th ed. Philadelphia: Saunders (2004).

[17] World Health Organization (WHO) Global Database on Body Mass Index, BMI classification [Internet]. Geneva (2006). Available online at: http://apps.who.int/bmi/index.jsp?introPage=intro_3.html (accessed May 27, 2020).

[18] World Health Organization (WHO). Waist circumference and waist–Hip ratio: report of a WHO expert consultation [Internet]. Geneva (2008). Available online at: https://apps.who.int/iris/bitstream/handle/10665/44583/9789241501491_eng.pdf;jsessionid=D961239D259944D3E13149E1FE4D8B6F?sequence=1 (accessed May 27, 2020).

[19] World Health Organization (WHO) Online Q&A on hypertension [Internet]. Geneva (2015). Available online at: https://www.who.int/features/qa/82/en/ (accessed May 27, 2020).

[20] World Health Organization (WHO) Use of Glycated Haemoglobin (HbA1c) in the Diagnosis of Diabetes Mellitus [Internet]. Geneva (2011). Available online at: https://apps.who.int/iris/bitstream/handle/10665/70523/WHO_NMH_CHP_CPM_11.1_eng.pdf?sequence=1&isAllowed=y (accessed May 27, 2020).

[21] American Diabetes Association Classification and diagnosis of diabetes: standards of medical care in diabetes. Diabetes Care. (2019) 42(Suppl 1):S13–28. 10.2337/dc19-S002 30559228

[22] Pearson TA Mensah GA Alexander RW Anderson JL Cannon RO Criqui M. Markers of inflammation and cardiovascular disease: application to clinical and public health practice: a statement for healthcare professionals from the centers for disease control and prevention and the American Heart Association. Circulation. (2003). 107:499–511. 10.1161/01.CIR.0000052939.59093.45 12551878

[23] Ampath Ampath desk reference: guide to laboratory tests. 2nd ed. Centurion: Ampath (2016). Available online at: https://www.ampath.co.za/pdfs/Desk-Reference-web.pdf (accessed May 26, 2020).

[24] Forget P Khalifa C Defour J-P Latinne D Van Pel M-C De Kock M. What is the normal value of the neutrophil-to-lymphocyte ratio? BMC Res Notes. (2017) 10:12. 10.1186/s13104-016-2335-5 28057051 PMC5217256

[25] Statistics South Africa Mortality and causes of death in South Africa, 2016: Findings from death notification. Pretoria (2018). Available online at: http://www.statssa.gov.za/publications/P03093/P030932016.pdf (accessed May 08, 2019).

[26] Stringhini S Forrester TE Plange-Rhule J Lambert EV Viswanathan B Riesen W. The social patterning of risk factors for noncommunicable diseases in five countries: evidence from the modeling the epidemiologic transition study (METS). BMC Public Health. (2016) 16:956. 10.1186/s12889-016-3589-5 27612934 PMC5017030

[27] Wells JC. The capacity-load model of non-communicable disease risk: understanding the effects of child malnutrition, ethnicity and the social determinants of health. Eur J Clin Nutr. (2018) 72:688–97. 10.1038/s41430-018-0142-x 29748656

[28] Gray A Vawda Y Health policy and legislation. In: Padarath A Barron P editors. South African Health Review 2017. Durban: Health Systems Trust (2017).

[29] Shisana O Labadarios D Rehle T Simbayi L Zuma K Dhansay A South African National Health and Nutrition Examination Survey (SANHANES-1). Cape Town: HSRC Press (2014).

[30] Wu F Guo Y Chatterji S Zheng Y Naidoo N Jiang Y. Common risk factors for chronic non-communicable diseases among older adults in china, ghana, mexico, india, russia and south africa: the study on global ageing and adult health (SAGE) wave 1. BMC Public Health. (2015) 15:88. 10.1186/s12889-015-1407-0 25885218 PMC4335695

[31] Walsh CM van Rooyen FC. Household food security and hunger in rural and urban communities in the free state province, South Africa. Ecol Food Nutr. (2015) 54:118–37. 10.1080/03670244.2014.964230 25551521

[32] Maimela E. Alberts M, Modjadji SE, Choma SS, Dikotope SA, Ntuli TS, et al. The prevalence and determinants of chronic non-communicable disease risk factors amongst adults in the dikgale health demographic and surveillance system (HDSS) site, limpopo province of South Africa. PLoS ONE. (2016) 11:e0147926. 10.1371/journal.pone.0147926 26882033 PMC4755539

[33] Tydeman-Edwards R Van Rooyen FC Walsh CM. Obesity, undernutrition and the double burden of malnutrition in the urban and rural southern free state, South Africa. Heliyon. (2018) 4:e00983. 10.1016/j.heliyon.2018.e00983 30534616 PMC6278724

[34] Steyn NP McHiza ZJ. Obesity and the nutrition transition in Sub-Saharan Africa. Ann N Y Acad Sci. (2014) 1311:88–101. 10.1111/nyas.12433 24725148

[35] Steyn K Levitt NS Health services research in South Africa for chronic diseases of lifestyle. In: Steyn K Fourie J Temple N editors. Chronic diseases of lifestyle in South Africa: 1995–2005. Technical report. Cape Town: South African Medical Research Council (2006).

[36] National Department of Health Statistics South Africa (Stats SA), South African Medical Research Council (SAMRC), and ICF. 2019 South Africa Demographic and Health Survey 2016 [Internet]. Pretoria, South Africa, and Rockville, Maryland, USA: NDoH, Stats SA, SAMRC, and ICF (2018). Available online at: https://dhsprogram.com/pubs/pdf/FR337/FR337.pdf (accessed May 08, 2019).

[37] Egbujie BA Igumbor EU Puoane T. A cross-sectional study of socioeconomic status and cardiovascular disease risk among participants in the prospective urban rural epidemiological (PURE) study. S Afr Med J. (2016) 106:900–6. 10.7196/samj.2016.v106i9.10456 27601117

[38] National Department of Health Statistics South Africa (Stats SA), South African Medical Research Council (SAMRC), and ICF. South Africa Demographic and Health Survey 2016. Key Findings [Internet]. Pretoria, South Africa, and Rockville, Maryland, USA: NDoH, Stats SA, SAMRC, and ICF (2018). Available online at: https://dhsprogram.com/pubs/pdf/SR248/SR248.pdf (accessed May 08, 2019).

[39] Johannsen NM Swift DL Johnson WD Dixit VD Earnest CP Blair SN. Effect of different doses of aerobic exercise on total white blood cell (WBC) and WBC subfraction number in postmenopausal women: results from DREW. PLoS ONE. (2012) 7:e31319. 10.1371/journal.pone.0031319 22363616 PMC3281960

[40] Pereira MA Swain J Goldfine AB Rifai N Ludwig DS. Effects of a low-glycemic load diet on resting energy expenditure and heart disease risk factors during weight loss. JAMA. (2004) 292:2482–90. 10.1001/jama.292.20.2482 15562127

[41] Liu S Willett WC Stampfer MJ Hu FB Franz M Sampson L. A prospective study of dietary glycemic load, carbohydrate intake, and risk of coronary heart disease in US women. Am J Clin Nutr. (2000) 71:1455–61. 10.1093/ajcn/71.6.1455 10837285

[42] Mazurek KL Zmijewski P Czajkowska A Lutosławska G High-sensitivity c-reactive protein (hscrp) in young adults: relation to aerobic capacity, physical activity and risk factors for cardiovascular diseases. Biol Sport. (2011) 28:227–32. 10.5604/965482

[43] Zarzour W Dehneh N Rajab M. High-sensitive c-reactive protein levels in a group of syrian university male students and its associations with smoking, physical activity, anthropometric measurements, and some hematologic inflammation biomarkers. Int J Inflam. (2017) 2017:7326527. 10.1155/2017/7326527 28487812 PMC5402232

[44] Azab B Chainani V Shah N McGinn JT. Neutrophil-lymphocyte ratio as a predictor of major adverse cardiac events among diabetic population: a 4-year follow-up study. Angiology. (2013) 64:456–65. 10.1177/0003319712455216 22904109

[45] Emerging Risk Factors Collaboration Kaptoge S Di Angelantonio ED Pennells L Wood AM White IR. C-reactive protein, fibrinogen, and cardiovascular disease prediction. N Engl J Med. (2012) 367:1310–20. 10.1056/NEJMoa1107477 23034020 PMC3714101

[46] Zhang Y Gu Y Wang N Qi Z Ng N Wang R Association between anthropometric indicators of obesity and cardiovascular risk factors among adults in Shanghai, China. BMC Public Health. (2019) 19:1035 10.1186/s12889-019-7366-0 31375086 PMC6679475

[47] Mekonnen T Animaw W Seyum Y. Overweight/obesity among adults in north-western ethiopia: a community-based cross-sectional study. Arch Public Health. (2018) 76:18. 10.1186/s13690-018-0262-8 29515803 PMC5836379

[48] Norman R Bradshaw D Steyn K Chronic diseases, risk factors and lifestyles based on the South African adult demographic and health survey. In: Bradshaw D Steyn K editors. Poverty and chronic diseases in South Africa. Tygerberg: Burden of Diseases Research Unit (2001).

[49] Zekewos A Egeno T Loha E. The magnitude of hypertension and its risk factors in southern Ethiopia: a community based study. PLoS ONE. (2019) 14:e0221726. 10.1371/journal.pone.0221726 31461475 PMC6713347

[50] Mbouemboue OP Ngoufack TJ. High blood pressure prevalence, awareness, control, and associated factors in a low-resource african setting. Front Cardiovasc Med. (2019) 6:119. 10.3389/fcvm.2019.00119 31544107 PMC6728810

[51] Mahmood SE Ahmad A Kashyap S Prevalence and predictors of hypertension among adults of urban Lucknow, India: a community-based study. Heart India. (2019) 7:43–8. 10.4103/heartindia.heartindia_6_19

[52] Vijayakumar G Manghat S Vijayakumar R Simon L Scaria LM Vijayakumar A. Incidence of type 2 diabetes mellitus and prediabetes in kerala, india: results from a 10-year prospective cohort. BMC Public Health. (2019)19:140. 10.1186/s12889-019-6445-6 30704495 PMC6357479

[53] American Diabetes Association prevention or delay of type 2 diabetes: standards of medical care in diabetes. Diabetes Care. (2019) 42:S29–33. 10.2337/dc19-S003 30559229

[54] Baird J Jacob C Barker M Fall CH Hanson M Harvey NC. Developmental origins of health and disease: a lifecourse approach to the prevention of non-communicable diseases. Healthcare (Basel). (2017) 5:14. 10.3390/healthcare5010014 28282852 PMC5371920

[55] Mikkelsen B Williams J Rakovac I Wickramasinghe K Hennis A Shin H-R. Life course approach to prevention and control of non-communicable diseases. BMJ. (2019) 364:l257. 10.1136/bmj.l257 30692103 PMC6349133

